# Biobased Composites by Photoinduced Polymerization of Cardanol Methacrylate with Microfibrillated Cellulose

**DOI:** 10.3390/ma15010339

**Published:** 2022-01-04

**Authors:** Alessandra Vitale, Samantha Molina-Gutiérrez, W. S. Jennifer Li, Sylvain Caillol, Vincent Ladmiral, Patrick Lacroix-Desmazes, Sara Dalle Vacche

**Affiliations:** 1 Department of Applied Science and Technology, Politecnico di Torino, 10129 Torino, Italy; alessandra.vitale@polito.it; 2 Research Unit, INSTM—Politecnico di Torino, 50121 Florence, Italy; 3 ICGM, University Montpellier, CNRS, ENSCM, 34095 Montpellier, France; samantha.molinag@gmail.com (S.M.-G.); jenli87@gmail.com (W.S.J.L.); sylvain.caillol@enscm.fr (S.C.); vincent.ladmiral@enscm.fr (V.L.); patrick.lacroix-desmazes@enscm.fr (P.L.-D.)

**Keywords:** photoinduced polymerization, biobased composites, cardanol methacrylate, microfibrillated cellulose, biobased polymers

## Abstract

Biobased monomers and green processes are key to producing sustainable materials. Cardanol, an aromatic compound obtained from cashew nut shells, may be conveniently functionalized, e.g., with epoxy or (meth)acrylate groups, to replace petroleum-based monomers. Photoinduced polymerization is recognized as a sustainable process, less energy intensive than thermal curing; however, cardanol-based UV-cured polymers have relatively low thermomechanical properties, making them mostly suitable as reactive diluents or in non-structural applications such as coatings. It is therefore convenient to combine them with biobased reinforcements, such as microfibrillated cellulose (MFC), to obtain composites with good mechanical properties. In this work a cardanol-based methacrylate monomer was photopolymerized in the presence of MFC to yield self-standing, flexible, and relatively transparent films with high thermal stability. The polymerization process was completed within few minutes even in the presence of filler, and the cellulosic filler was not affected by the photopolymerization process.

## 1. Introduction

Sustainable polymer-based materials may be conveniently prepared through the photoinduced curing of biobased monomers [1,2,3]. Photoinduced polymerization is widely recognized as a green technology, thanks to its low energy requirements, high reaction rates at room temperature, no need for solvents, and low VOC emissions [4,5]. Among the various biobased monomers explored for this application are those derived from vegetable oils such as soybean or linseed oils, from lignin such as vanillin or eugenol, from terpenes, from itaconic acid and succinic acid, etc. [6,7]. UV and two-photon polymerization of biocompatible and appropriately functionalized biobased monomers and oligomers derived from fatty acids, polylactic acid, alginate microgel, hyaluronic acid, chitosan, hydroxypropyl methylcellulose, and gelatin, were, e.g., used for injectable viscous materials for implants and hydrogels [8].

However, photocured biobased polymers often have low thermomechanical properties, which are only suitable for non-structural applications such as coatings or adhesives, and they are mostly used in combination with fossil-based co-monomers or reinforcements [5]. The addition of natural fillers, such as microfibrillated cellulose, to biobased photocurable monomers is thus particularly interesting for preparing fully biobased composites.

Cardanol, particularly, is an interesting aromatic compound obtained by thermal treatment of cashew nutshell liquid (CNSL) extracted from the shell of the cashew seed. This nonedible by-product of the cashew nut industry is a commercially abundant and inexpensive renewable resource. Cardanol is a phenolic lipid with a C15 side chain that can be saturated (4%), mono-olefinic (41%), di-olefinic (19%), or tri-olefinic (35%), leading to an average of two C=C double bonds per molecule [9]. In the polymer field, cardanol is very appealing as its multiple reactive sites, i.e., the phenolic group and the unsaturations of the side chain, can be exploited for derivatization or functionalization [10]. Epoxidized cardanol derivatives were polymerized through photoinduced cationic polymerization reactions [11]; they were also used for the production of biobased composites with cellulose microfibrils; while composites with good overall properties could be prepared, a drawback of photoinduced cationic polymerization is the degradation of the cellulosic filler by the acid species generated in the reaction, leading to lower thermal stability of the composites [12,13].

(Meth)acrylated compounds can be polymerized through a radical mechanism [14], which could be an advantage when a cellulosic filler is present. Specifically, acrylates derived from cardanol were successfully used for radical polymerization reactions. Conventional radical polymerization of cardanyl acrylate was found to produce crosslinked materials. A linear polymer was obtained by solution polymerization of cardanyl acrylate in toluene using azobisisobutyronitrile (AIBN) as the initiator. Upon removal of solvent, the polymer underwent crosslinking upon exposure to air (or UV light), resulting in an insoluble transparent film. In bulk and suspension polymerization, the polymer underwent in situ crosslinking even in the absence of any crosslinking agent [15]. Crosslinked copolymers of cardanyl acrylate with vinyl monomers were also obtained by conventional radical polymerization in bulk or suspension polymerization [16,17]. The synthesis of linear poly(cardanyl acrylate) completely soluble in THF could be instead carried out by ATRP in bulk at 95 °C, with a conversion of 62% [18].

The use of cardanol-based acrylates and epoxidized cardanol-based acrylates as a reactive diluent in photocurable castor oil polyurethane acrylate and acrylated epoxidized soybean oil resins was also reported [19,20,21]. Cardanol methacrylate was used as additive for UV-curable tripropylene glycol diacrylate/1,6-hexanediol diacrylate formulations, to increase their hydrophobicity [22]. Acrylated cardanol diphenyl phosphate was added to urethane acrylate UV-curable coatings, enhance flame-retardant properties, and reduce volume shrinkage [23,24]. Finally, cardanol-based multi-arm acrylates, in which the acrylate groups are attached to the alkyl side chain of the cardanol moieties, were synthesized and used to produce UV-curable coatings [25,26,27,28,29].

To the best of our knowledge, the photoinduced radical polymerization of cardanol methacrylate in the presence of a cellulosic filler has not yet been reported. In this work we prepared composite films by the photopolymerization of cardanol methacrylate reinforced with microfibrillated cellulose. The photoinduced polymerization reaction reached a high degree of conversion within minutes, allowing the obtainment self-standing flexible films.

## 2. Materials and Methods

### 2.1. Materials

**Figure 1:**
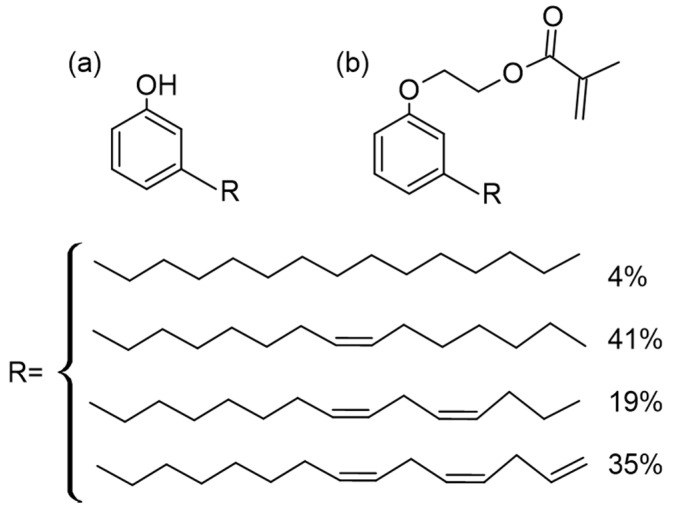
Structures of (**a**) cardanol and (**b**) cardanol methacrylate (CM).

A cardanol-based methacrylate monomer, from here on simply called cardanol methacrylate (CM), was derived from cardanol (Figure 1) with the one-pot, two-step synthesis described in [7]. For the synthesis, Cardanol NX2026 containing 1% cardol was kindly supplied by Cardolite; ethylene carbonate (98%, Sigma-Aldrich, St. Quentin Fallavier, France), 1,5-diazabicyclo[4.3.0]non-5-ene (98%, Sigma-Aldrich, St. Quentin Fallavier, France), triethylamine (99.5%, Sigma-Aldrich, St. Quentin Fallavier, France), methacrylic anhydride (94%, Sigma-Aldrich, St. Quentin Fallavier, France), sodium hydroxide (NaOH, 98%, Sigma-Aldrich, St. Quentin Fallavier, France), ethyl acetate (>99%, VWR, Rosny sous bois, France), and sodium sulfate (Na_2_SO_4_, >99%, Sigma-Aldrich, St. Quentin Fallavier, France), were used as received; dichloromethane was dried with molecular sieves (4 Å, 2.5–5.0 mm beads (4–8 Mesh), Fisher Chemical, Illkirch, France) and kept under N_2_ before use; deionized water (1 μS cm^−1^) was obtained using a D8 ion exchange demineralizer (A2E Affinage de L’Eau, Mauguio, France).

**Figure 2:**
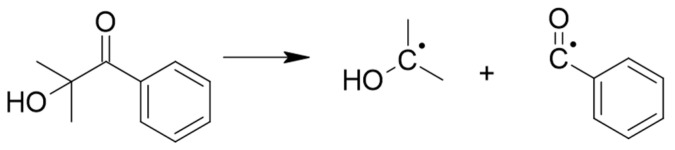
Homolytic cleavage of 2-hydroxy-2-methylpropiophenone [30].

The radical photoinitiator (PI) used in this work was 2-hydroxy-2-methylpropiophenone (Darocur 1173, BASF, Ludwigshafen, Germany), a Norrish Type I photoinitiator that, when irradiated with UV light, undergoes homolytic cleavage, yielding two carbon-centered radicals, as depicted in Figure 2.

Wood-derived microfibrillated cellulose (MFC), in the form of a paste with 10 wt% solids in water (Exilva F01-V), was kindly provided by Borregaard (Sarpsborg, Norway). For the preparation of composites via the solvent exchange technique, acetone (≥99.5% Sigma-Aldrich S.r.l., Milan, Italy), was used.

### 2.2. Preparation of the Photocurable Resin and Composites

The photocurable resin was prepared by adding the radical photoinitiator (PI) to cardanol methacrylate (CM) and mixing for 5 min over a magnetic stirrer (AREX Hot Plate Stirrer, VELP Scientifica, Usmate (MB), Italy). The concentration of PI in the photocurable resin formulation (CM + PI) was 2–3 wt%. To prepare the composite films, the MFC 10 wt% paste was diluted with deionized water and dispersed with a homogenizer (Ultraturrax T10, IKA^®^-Werke GmbH & Co. KG, Staufen, Germany) at about 20k rpm, for 5 min, to obtain a suspension with a solid content of 0.75 wt%. The MFC suspension was then filtered with a Büchner funnel connected to vacuum, fitted with a 47-mm-diameter Durapore^®^ membrane filter (hydrophilic PVDF, 0.65-micrometer pore size, Merck Millipore, Darmstadt, Germany). The wet mats of cellulosic fibers formed on the filter were transferred to an acetone bath, allowing the exchange of water with acetone. The solvent was refreshed twice over 24 h. For the impregnation, the photocurable CM + PI resin was diluted with acetone to obtain solutions containing either 15 wt% or 30 wt% of resin; the mats were then transferred in a bath containing the selected solution of the photocurable resin. The impregnation time was set at 2.5 h in the dark to prevent any reaction. Finally, the impregnated mats were removed from the bath, and the solvent was evaporated at room temperature under vacuum for about 10 min, obtaining the uncured composites, which were stored at 4 °C in the dark until further use. The final resin and MFC weight fractions in the composites were estimated gravimetrically from the weight of MFC used for preparing the mat and the final weight of the composite after solvent evaporation. The composites prepared using the solution with 30 wt% of resin contained approximately 25 wt% MFC and will be designated as low MFC (L-MFC) composites; those prepared using the solution with 15 wt% of resin contained approximately 45 wt% MFC and will be designated as high MFC (H-MFC) composites.

### 2.3. Photoinduced Polymerization of the Resin and Composites

Real-time Fourier transform infrared (rt-FTIR) spectroscopy was used to monitor the photoinduced polymerization of the CM + PI resin using a Nicolet iS50 spectrometer (Thermo Fisher Scientific Inc., Waltham, MA, USA). The equipment was fitted with a custom-made accessory allowing in situ irradiation of horizontal samples and with a high-pressure mercury–xenon lamp Lightning Cure LC8 equipped with a flexible light guide (L9566-02A, 220 to 600 nm) by Hamamatsu (Hamamatsu Photonics Italia S.R.L, Arese (MI), Italy). The intensity of the UV light was measured with an EIT Powerpuck^®^ II radiometer (EIT, LLC., Leesburg, VA, USA) and was set at 218 ± 13 mW cm^−2^ (UVA + UVB + UVC). For the analysis, resin samples were spread on silicon wafers by a 10-micrometer wire-wound applicator; they were then irradiated in situ during the rt-FTIR analysis for up to 9 min, either under air or protected from air with a 30-micrometer-thick polypropylene (PP) film. The spectra were acquired in transmission mode, in the 650–4000 cm^−1^ range, with 1 scan per spectrum and a resolution of 4 cm^−1^, with a sampling interval of 0.36 s.

In order to assess the advancement of the polymerization reaction of the resin and of the composites under inert atmosphere (nitrogen), a 5000-EC UV flood lamp system (Dymax Corporation, Torrington, CT, USA) with a medium intensity mercury bulb (320–390 nm) was used for the photopolymerization; the lamp was fitted with a chamber connected with a nitrogen flow and equipped with a quartz window through which the specimens were exposed to the UV light (see Figure S1). The UV intensity (UVA) was checked by means of a UV Power Puck II radiometer (EIT, LLC., Leesburg, USA) and was fixed at 100 ± 2, 130 ± 2 or 140 ± 2 mW cm^−2^ by setting the distance between the specimen and the light source. The resins were spread by 10-micrometer wire-wound bar applicators on silicon wafers or on glass slides by a 50-micrometer wire-wound bar applicator. The composites were in the form of self-standing films; the L-MFC ones were 140 μm thick, and the H-MFC ones were 90 μm thick. The resin coatings were irradiated only on the free side, while the irradiation of the composites was carried out by turning the sample upside down at given intervals to have homogeneous irradiation on the two sides. The resin coatings were analyzed by Fourier transform infrared spectroscopy with a Nicolet iS50 spectrometer (Thermo Fisher Scientific Inc., Waltham, MA, USA). Transmission mode was used to analyze the 10-micrometer-thick resin coatings on silicon wafers after different irradiation times, in the 400–4000 cm^−1^ range, with 32 scans per spectrum and a resolution of 4 cm^−1^; polymerized resin samples, detached from the glass slides, were analyzed in attenuated total reflectance (ATR) mode using an ATR-Smart Orbit accessory with a diamond crystal in the 525–4000 cm^−1^ range, with 32 scans per spectrum and a resolution of 4 cm^−1^. The composites were analyzed in ATR mode, with a Nicolet 5700 FTIR spectrometer equipped with an ATR-Smart Orbit accessory with a diamond crystal (ThermoElectron Corp., Milan, Italy); the spectra were acquired in the 525–4000 cm^−1^ range, with 64 scans per spectrum and a resolution of 2 cm^−1^.

### 2.4. Characterization Methods

The conversion of double bonds was calculated from the FTIR spectra using the following Equation (1):

(1) $$Conversion_{t=x}\left(\%\right)=100*\left(1-\frac{\frac{A_{t=x}}{Ref.A_{t=x}}}{\frac{A_{t=0}}{Ref.A_{t=0}}}\right)$$

where *Ref. A* is the absorbance estimated as area of the reference peak centered at 1583 cm^−1^ corresponding to the aromatic C–C (in ring) stretching, and *A* is the absorbance estimated as area of the peak corresponding to the monitored functional group. Specifically, the conversion of methacrylate double bonds was monitored using the band at 1638 cm^−1^ and the conversion of the side chain double bonds using the band at 3008 cm^−1^.

The insoluble fractions of the composites were assessed by measuring the weight of the samples, wrapped in a fine metallic mesh, before and after immersion in toluene for 24 h and evaporation of the residual solvent at room temperature for 24 h followed by drying at 70 °C for 1 h.

Thermogravimetric analysis (TGA) was performed using a TG 209 F1 Libra thermobalance (NETZSCH-Gerätebau GmbH, Selb, Germany). Scans were made from 50 °C to 700 °C with a heating rate of 10 °C min^−1^, under a N_2_ flux, to prevent thermo-oxidative processes. The first derivative of the weight curve (DTG) was calculated in order to better resolve the main thermal decomposition steps of the analyzed materials.

Dynamic mechanical analysis (DMA) was performed with a Q800 (TA Instruments—Waters SpA, Sesto San Giovanni (MI), Italy), in the tensile mode. The temperature was increased from −90 °C to 120 °C at a rate of 3 °C min^−1^. The frequency was set at 1 Hz, and the strain was set at 0.1%. The specimens had a length of approximately 10 mm between the clamps and a width of approximately 5 mm.

## 3. Results

### 3.1. Photoinduced Polymerization of Cardanol Methacrylate and Its Composites with MFC

**Figure 3:**
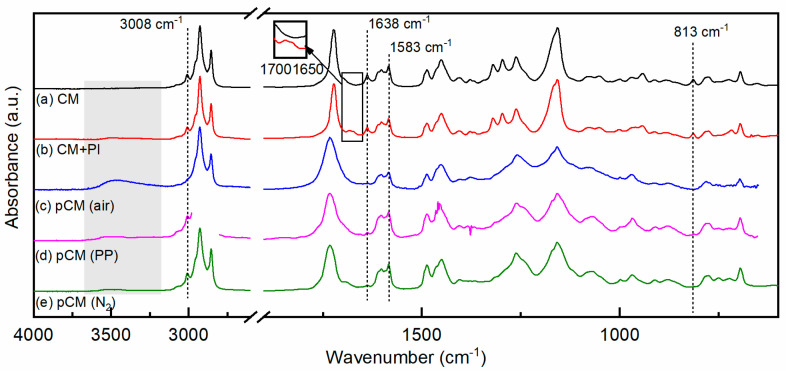
FTIR spectra acquired in transmission mode of: (**a**) cardanol methacrylate, CM; (**b**) cardanol methacrylate with photoinitiator, CM + PI, and (**c**–**e**) poly(cardanol methacrylate), pCM, photopolymerized under air, protected by a PP film or under nitrogen (N_2_). In the spectrum (**d**) the signal in the 2980–2800 cm^−2^ region is saturated due to the presence of the PP film.

The FTIR spectrum of CM is shown in Figure 3a. The absence in the spectrum of a broad absorption band above 3100 cm^−1^ typical of hydroxyls confirmed the replacement of the phenolic OH groups of cardanol by methacrylate groups, which instead gave rise to peaks corresponding to C=O and C=C stretching vibrations at 1723 cm^−1^ and 1638 cm^−1^, respectively, and to C=CH_2_ twisting at 813 cm^−1^ [9]. Characteristic vibrations of the alkyl chain were found in the 3100–2800 cm^−1^ region, where the peak at 3008 cm^−1^ was attributed to the stretching of =C–H *cis* bonds [20,31,32,33,34], and two intense peaks at 2927 cm^−1^ and 2854 cm^−1^ corresponded to sp^3^ C–H bond vibrations in –CH_3_ and –CH_2_ groups, respectively [20,35,36]. The aromatic ring was characterized by the vibrations of the C=C aromatic bonds in the 1550–1600 cm^−1^ region, with two strong peaks at 1601 cm^−1^ and 1583 cm^−1^ [20,33,36] and by peaks characteristic of the meta substituted aromatic rings vibrations (670–710, 750–805, 870–900 cm^−1^).

The FTIR spectrum of 2-hydroxy-2-methylpropiophenone (PI) in its undissociated form (i.e., before irradiation) has a strong band centered around 1680 cm^−1^ [37]; indeed, this band appeared in the spectrum of the non-irradiated CM + PI formulation, as shown in Figure 3b, in the 1690–1660 cm^−1^ region (enlarged in the inset in Figure 3).

**Figure 4:**
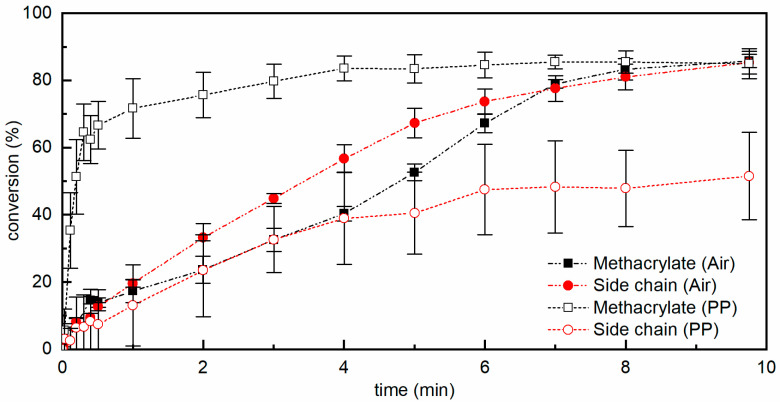
Conversion as a function of time of methacrylate double bonds (squares) and of side chain double bonds (circles) for cardanol methacrylate resins containing PI, irradiated with a light intensity of 218 mW cm^−2^ in the UVA–UVC range under air (full symbols) and protected from air by means of a PP film (hollow symbols); lines connect the experimental points and are only a guide for the eyes.

The photoinduced polymerization of cardanol methacrylate in the presence of PI proceeds via a radical mechanism that is affected by oxygen [38,39,40]; therefore, its kinetics was studied by real-time FTIR both under air and protected by air by means of a PP film. The FTIR spectra of pCM samples obtained by photopolymerization in air and protected by a PP film are shown in Figure 3c,d, respectively. The percentages of reacted methacrylate double bonds and side chain double bonds (conversion) as a function of irradiation time are reported in Figure 4. They were estimated, using Equation (1) (as explained in Section 2.4), from the absorbances of the bands at 1638 cm^−1^ and 3008 cm^−1^, respectively, in the FTIR spectra taken at different times of irradiation.

When the resins were irradiated under air the reaction of the methacrylate double bonds started slowly, and 50% conversion was reached after approximately 5 min; then the reaction rate increased, and a conversion of 85% was reached within the 9 min of irradiation. The slow initial rate of reaction was attributed to oxygen inhibition, typical for (meth)acrylate resins [39,40]. Indeed, oxygen can scavenge the initiating free radicals and the growing polymer radicals, forming peroxyl radicals; these are not reactive towards (meth)acrylate double bonds, and thus the polymerization reaction slows down until oxygen is consumed [30]. The peroxyl radicals, particularly when ether groups are present on the growing chain, may then form hydroperoxides, abstracting hydrogen atoms from the polymer backbone and thus originating a carbon-centered polymer radical that can react with the (meth)acrylate monomers, causing the re-initiation of the polymerization reaction [41]; this gives rise to a typical sigmoidal curve for the conversion as a function of time. Confirming this type of mechanism, a band centered around 3400 cm^−1^, characteristic of hydroperoxides, appeared [30,42], as shown in the area shaded in gray in Figure 3. At the same time, the peak centered at 3008 cm^−1^ decreased, indicating that the unsaturations of the alkyl side chain were consumed; almost 70% of the side chain double bonds were consumed after 5 min of irradiation, and 85% by the end of the irradiation time (9 min). This also suggests the presence of the autoxidation of the side chain of cardanol, with a mechanism similar to that of unsaturated fatty acids in the presence of oxygen; peroxyl radicals abstract a hydrogen from the side chain, forming hydroperoxides, which then further react with other species, creating crosslinks or carbonyl groups [15,42]. This was further confirmed as the band characteristic of carbonyl groups broadened and became more intense.

When the CM + PI resin was irradiated while being covered by the PP film and thus protected from air, the conversion as a function of time of the methacrylate double bonds and of side chain double bonds was very different. In the case of methacrylate double bonds, the polymerization proceeded rapidly since the start of irradiation, reaching more than 50% conversion within 30 s, and a conversion higher than 80% was reached within about 4 min, slowly increasing to more than 85% by the end of the 9 min of irradiation. Some variation in the initial reaction rate was observed within different measurements, possibly due to residual oxygen entrapped in the resin coating or between the resin and the PP protection. Indeed, when the polymerization proceeded more slowly, a larger decrease in the peak at 3008 cm^−1^ and rise of the band at 3400 cm^−1^ could be detected, indicating the presence of residual oxygen. The consumption of the side chain double bonds, through the autoxidation mechanism requiring the presence of oxygen, was very limited for short irradiation times compared to irradiation under air; however, it had still reached approximately 50% by the end of the irradiation.

**Figure 5:**
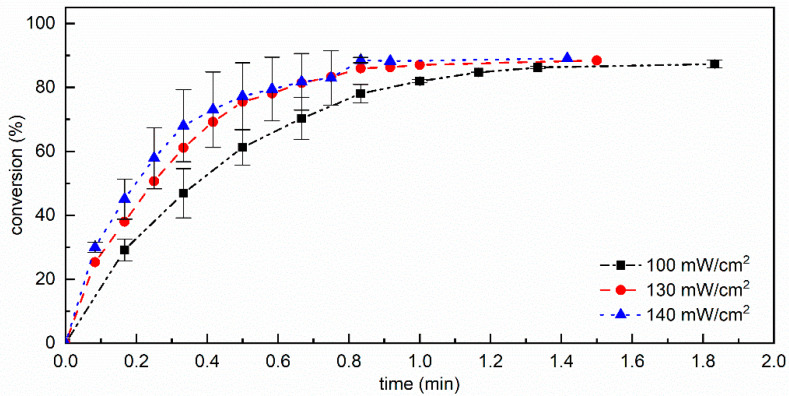
Conversion of methacrylate double bonds as a function of time for cardanol methacrylate resins containing photinitiator (CM + PI), cured at 100, 130, and 140 mW cm^−2^ in the UVA range under nitrogen; lines connect the experimental points and are only a guide for the eyes.

To guarantee a completely oxygen-free environment, a series of measurements was performed using a UV flood lamp equipped with a nitrogen-purged chamber. The CM + PI resin was irradiated under nitrogen for given time intervals and immediately analyzed by FTIR spectroscopy in transmission. Three light intensities were used, namely 100, 130, and 140 mW cm^−2^, in the UVA range. The spectrum of pCM obtained by photopolymerization under a nitrogen atmosphere 100 mW cm^−2^ is given in Figure 3e and is representative of the spectra obtained at all three light intensities. For reference, cardanol methacrylate (CM) was irradiated in the absence of photoinitiator with UVA light under nitrogen for up to 80 s at 130 mW cm^−2^, and no changes were observed in the FTIR spectrum with respect to the non-irradiated CM. After the irradiation of the photocurable CM + PI resin, no change in the FTIR spectrum was detected above 2000 cm^−1^ for any of the three light intensities, confirming that irradiation with UVA in the absence of oxygen did not affect the alkyl side chain and that no hydroperoxides were produced. On the other hand, as expected, the peaks corresponding to the vibrations of the methacrylate group changed upon irradiation. The absorbance of the peaks at 1638 cm^−1^ and 813 cm^−1^ decreased; the peak at 1723 cm^−1^ broadened and shifted towards 1732 cm^−1^, while its absorbance remained unchanged, as the photopolymerization proceeded through the reaction of methacrylate C=C double bonds. The conversion of methacrylate double bonds at different irradiation times was calculated using Equation1 from the absorbance of the peak at 1638 cm^−1^ and is reported in Figure 5.

The conversion rate increased with increasing light intensity as expected, and in all cases a conversion of 87–89% was obtained in times ranging from 1 to 2 min, thus very rapidly as no side reactions took place.

**Figure 6:**
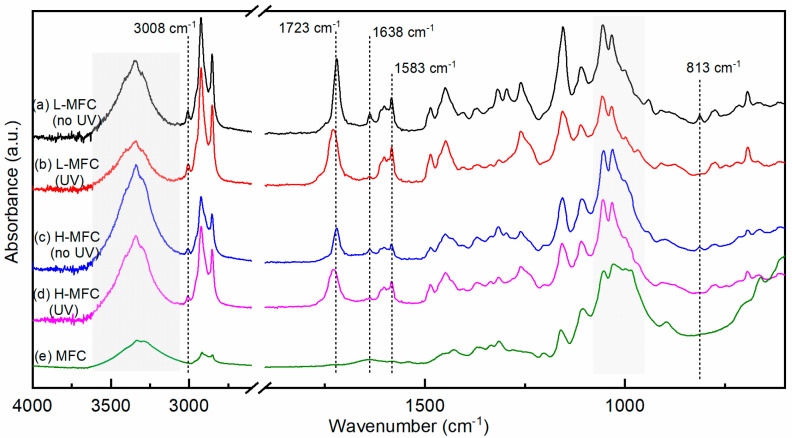
FTIR spectra acquired in ATR mode of: (**a**,**b**) L-MFC and H-MFC before irradiation (**c**,**d**) L-MFC and H-MFC after irradiation under nitrogen for 5 min at 100 mW cm^−2^; (**e**) dry MFC.

The composite materials were irradiated at 100 mW cm^−2^ under nitrogen; the FTIR spectra of the L-MFC and H-MFC composites before and after irradiation for 5 min are reported in Figure 6a–d, where the spectrum of dry MFC is also reported for reference.

**Figure 7:**
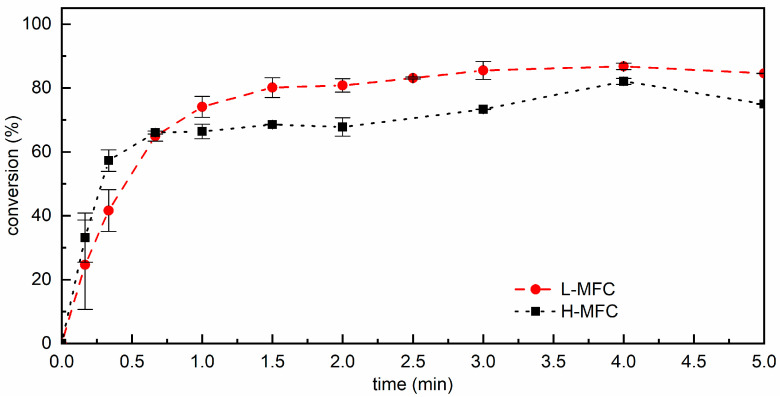
Conversion of methacrylate double bonds as a function of time for L-MFC and H-MFC composites irradiated with 100 mW cm^−2^ in the UVA range under nitrogen; lines connect the experimental points and are only a guide for the eyes.

The spectrum of dry MFC, shown in Figure 6e, was characterized by a broad band in the 3500–3000 cm^−1^ region corresponding to O–H stretching vibrations and relatively weak C–H stretching vibrations at 3000–2800 cm^−1^. A weak and broad peak centered at 1639 cm^−1^ was associated with water bound to the cellulose fibrils. Finally, intense bands in the fingerprint region were attributed to the C–O stretching of the pyranose ring skeletal vibration in the 1150–1030 cm^−1^ range, the C–O bending of cellulose alcohols involved in hydrogen bonds at 985 cm^−1^ [43], and the β-glycosidic bond vibration at 896 cm^−1^ [44,45,46,47,48,49]. The spectra of the uncured composites showed the main characteristic peaks of both MFC (highlighted in gray in Figure 6) and CM + PI resin; the change of the intensity of the peak at 985 cm^−1^ relative to the peaks in the 1150–1030 cm^−1^ range in the composite materials versus dry MFC may be ascribed to a decrease in hydrogen bonding involving cellulose alcohols. After irradiation under nitrogen, a decrease in the bands at 1638 cm^−1^ and 813 cm^−1^ was observed, while the peak at 1723 cm^−1^ broadened and shifted towards 1732 cm^−1^, while its absorbance remained unchanged, as observed also for pCM polymerized under nitrogen. It is important to notice that MFC absorbs light at wavelengths below 600 nm, and its absorbance increases with decreasing wavelength, particularly below 400 nm [50]; thus, it is expected to affect the photopolymerization reaction. The conversion of methacrylate double bonds as a function of time for the composites cured in inert atmosphere is shown in Figure 7. The reaction rates in the early stage of the photopolymerization were comparable within experimental variability for the two MFC fractions. While initial conversion rates were comparable for the composites and the resin, the polymerization rate of the composites decreased, more markedly when a higher amount of MFC was present, after 0.5 min. Thus, the final conversion was somewhat lower for the H-MFC composites (ca. 75–80%) than for the L-MFC composites (ca. 85%) and was reached within 3–5 min of irradiation.

### 3.2. Properties of the Photocured Resin and Its Composites

**Figure 8:**
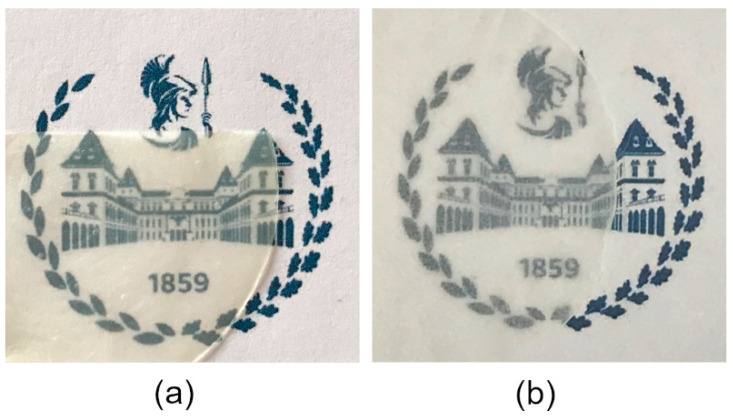
Photos of: (**a**) L-MFC composite, containing ca. 25 wt% MFC; (**b**) H-MFC composite containing ca. 45 wt% MFC.

The non-irradiated CM + PI resin had a slightly yellow hue, while cured resin coatings were clear and transparent. The MFC used in this work was a commercial product that has been fully characterized by Berglund and coworkers [51,52]; it consists of an entangled network of fibrils with a polydisperse size distribution, mainly having widths ranging from 20 nm to 1 μm, and lengths of approximately 5 to 40 μm, with a small fraction of coarser micrometer-sized fibrils with lower aspect ratio. After photopolymerization, the composite films were flexible and relatively transparent, although slightly hazy due to the diffusive effects of the texture formed by the MFC at the surface, with a slightly yellowish hue that was more intense for higher fractions of resin. Images of cured composite films are shown in Figure 8, while optical micrographs of their surfaces are given in Figure S2.

The insoluble content of the composites was measured after immersion in toluene for 24 h; it was 92% and 86% for the L-MFC and H-MFC composites, respectively. Taking into account the estimated weight fraction of cellulose, it was calculated that ca. 89% and 74% of the resin, for the L-MFC and H-MFC composites, respectively, remained insoluble. The poor solubility of poly(cardanol methacrylate) when polymerized in the presence of MFC is not unexpected; in fact, acrylic polymers can be grafted onto cellulose (either handsheet or fibers), obtaining covalent links. The propagation of the acrylic monomer is initiated by different radicals formed onto cellulose either by the photoinitiator or by the direct effect of UV light irradiation [53,54].

**Figure 9:**
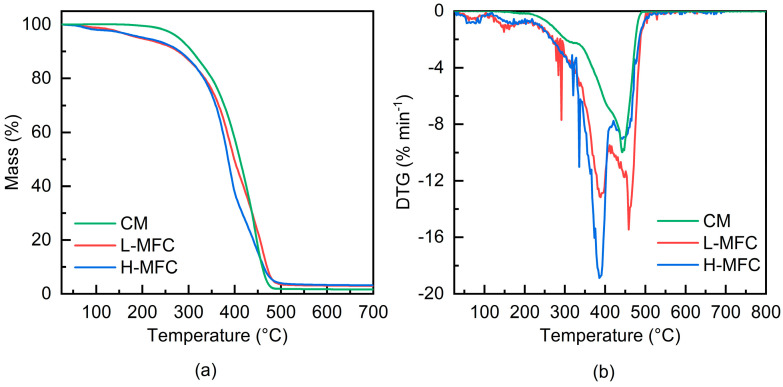
Thermogravimetric analysis results for cured CM resin, L-MFC, and H-MFC composites: (**a**) weight loss and (**b**) DTG curves.

The thermogravimetric curves obtained for pCM and L-MFC and H-MFC composites, all photopolymerized under nitrogen at 100 mW cm^−2^, are shown in Figure 9**,** together with the first derivative of the weight curves. As a reference, TGA analysis of the CM monomer and of L-MFC composite before polymerization are reported in Figure S3; TGA analysis of dry sheets made of the same MFC as used in this work can be found in [12]. The values of the T_d,5%_ and T_d,10%_ (i.e., the temperatures corresponding to a weight loss of the sample of 5% and 10%, respectively) and T_max_ (i.e., the temperatures corresponding to the main degradation peaks in the DTG curve) are summarized in Table S1. For the pCM resin, the T_d,5%_ was 280 °C, the T_max_ was 445 °C, and the residue was less than 2 wt%. The composites showed small weight losses at a low temperature, attributed to residual solvent or water evaporation; they had a T_d,5%_ = 205 °C owing to these initial losses. A first thermal decomposition event then followed with T_max_ at 388 °C attributed to MFC, and a second one with T_max_ at 445 °C and at 458 °C, for the H-MFC and the L-MFC composite, respectively, due to the CM resin component. The temperature at which the weight loss attributed to thermal decomposition of MFC occurred was slightly higher than that measured in our previous work for dry MFC sheets, i.e., 355 °C [12]; thus, not only did the radical photoinduced polymerization process not damage the MFC, but the presence of the resin even slightly retarded the thermal decomposition of the cellulosic reinforcement. Interestingly, this is the opposite of what was previously observed with cationic photoinduced polymerization, where the strong acid developed upon the scission of the photoinitiator was suspected to hydrolyze cellulose [12,13].

**Figure 10:**
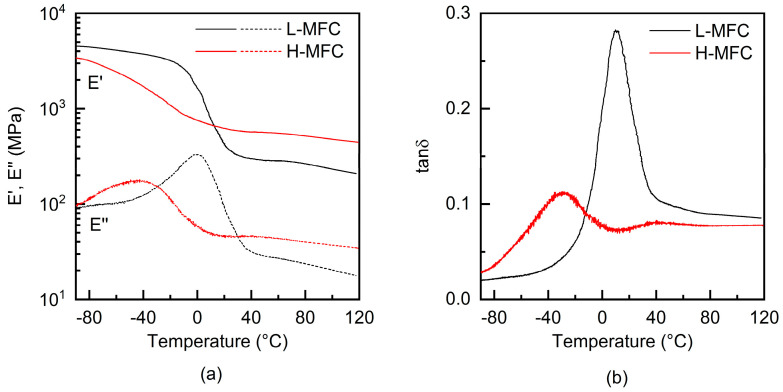
Dynamic mechanical analysis results for H-MFC and L-MFC composites, photopolymerized under nitrogen at 100 mW cm^−2^: (**a**) storage modulus E’ (solid lines) and loss modulus E’’ (dashed lines); (**b**) loss tangent, tanδ.

The viscoelastic properties of the photopolymerized composites were assessed by dynamic mechanical analysis (DMA); as a reference, DMA analysis of a dry MFC sheet is reported in Figure S4; the material showed no transitions in the examined temperature range and a storage modulus slowly and linearly decreasing with temperature from approximately 7 to 5 GPa. On the other hand, it was not possible to detach pCM from the substrate in the form of a self-standing film, as it was too soft, while the composites before UV irradiation, although in the form of a self-standing film, had too low mechanical properties and broke at the edges or were deformed while they were mounted in the DMA clamps. The storage (E’) and loss (E’’) moduli as a function of temperature of the L-MFC and H-MFC cured composites are reported in Figure 10a and the respective loss tangent plot (tanδ) in Figure 10b. The storage modulus (E’) curves showed a typical shape, with an initial slow E’ decrease with temperature and a step decrease at the glass transition temperature (T_g_) of the material, followed by a slow decrease. The L-MFC had a modulus in the glassy state of approximately 4.5 GPa, which in the rubbery state decreased to about 0.3 GPa; its tanδ curve showed a pronounced peak, indicating the T_g_ centered around 8 °C. These results are comparable with those obtained for composites prepared by photoinduced curing composites with an epoxidized cardanol matrix containing a similar wt% of MFC (of the same type as used in this work) [12]. For the H-MFC composite on the other hand, the storage modulus had a value of 3.4 GPa at −90 °C and decreased with temperature reaching in the rubbery state a value of 0.4 GPa; the lower decrease in the storage modulus was due to the higher amount of MFC reinforcement. The tanδ peak was centered around −28 °C. The lower T_g_ presented by the H-MFC composites is in agreement with results of the FTIR analysis; the high amount of cellulose hindering light transmission reduced the final conversion. The shoulder present around 50 °C in the tanδ was attributed to the evaporation of residual solvent [12].

## 4. Conclusions

The photoinduced polymerization of CM resins under UV light proceeded rapidly in the absence of oxygen, reaching 87–89% conversion of methacrylate double bonds within 1–2 min. When oxygen was present, the polymerization rate was initially slower; the consumption of the unsaturations on the alkyl chain and the formation of hydroperoxides were also observed. Nevertheless, when photoinduced polymerization was conducted under air a conversion of the methacrylate double bonds higher than 85% was reached within 9 min.

Composites containing microfibrillated cellulose were successfully photocured in the absence of oxygen, reaching conversions of methacrylate double bonds between 75% and 85% depending on the cellulose content. The composites were relatively transparent with a yellowish hue, and flexible; their glass transition temperature ranged from −28 °C to 8 °C. Their thermal stability was high, with a T_d,5%_ = 205 °C. Notably, the radical photopolymerization of the resin did not affect the thermal stability of the MFC in the composites, in contrast to what was previously observed with cationically photocured matrices.

Therefore, in this work self-standing flexible biobased composite films made of cardanol methacrylate and cellulosic fillers and characterized by good thermal and mechanical properties were easily produced by photoinduced radical polymerization.

## Data Availability

Raw FTIR data are available in the Zenodo repository (DOI: 10.5281/zenodo.5800056). Other data are available from the authors on request.

## Supplementary Materials

The following supporting information can be downloaded at: https://www.mdpi.com/article/10.3390/ma15010339/s1, Figure S1: Photo of (**a**) 5000-EC UV flood lamp system fitted with nitrogen-purged chamber and (**b**) nitrogen-purged chamber with sample inside; Figure S2: Optical micrographs of the surfaces of composite materials (dark field, in reflection mode); Figure S3: TGA analysis of (**a**) CM and (**b**) L-MFC composite before irradiation; Figure S4. DMA analysis of dry MFC sheet; Table S1: Main parameters from TGA and DMA measurements performed on dry MFC, pCM, and on photopolymerized L-MFC and H-MFC composites.
