# Supplementary material for: Biobased Composites by Photoinduced Polymerization of Cardanol Methacrylate with Microfibrillated Cellulose
Source: Materials (Basel). 2022 Jan 4;15(1):339. doi: 10.3390/ma15010339 (PMC8746221; doi:10.3390/ma15010339)
Supplement: Supplementary file 1 [file materials-15-00339-s001.zip › materials-1511604-supplementary.pdf]

Supplementary Information

Biobased composites by photoinduced polymerization of cardanol methacrylate with microfibrillated cellulose

Alessandra Vitale [1,2], Samantha Molina-Gutiérrez [3], W. S. Jennifer Li [3], Sylvain Caillol [3], Vincent Ladmiral [3], Patrick Lacroix-Desmazes [3] and Sara Dalle Vacche [1,2]*

[1] Department of Applied Science and Technology, Politecnico di Torino, 10129 Torino, Italy; alessandra.vitale@polito.it (A.V.)
[2] INSTM—Politecnico di Torino Research Unit, 50121 Firenze, Italy
[3] ICGM, Univ Montpellier, CNRS, ENSCM, 34000 Montpellier, France; sylvain.caillol@enscm.fr (S.C.), vincent.ladmiral@enscm.fr (V.L.), patrick.lacroix-desmazes@enscm.fr (P.L-D.)
* Correspondence: sara.dallevacche@polito.it (S.D.V.)

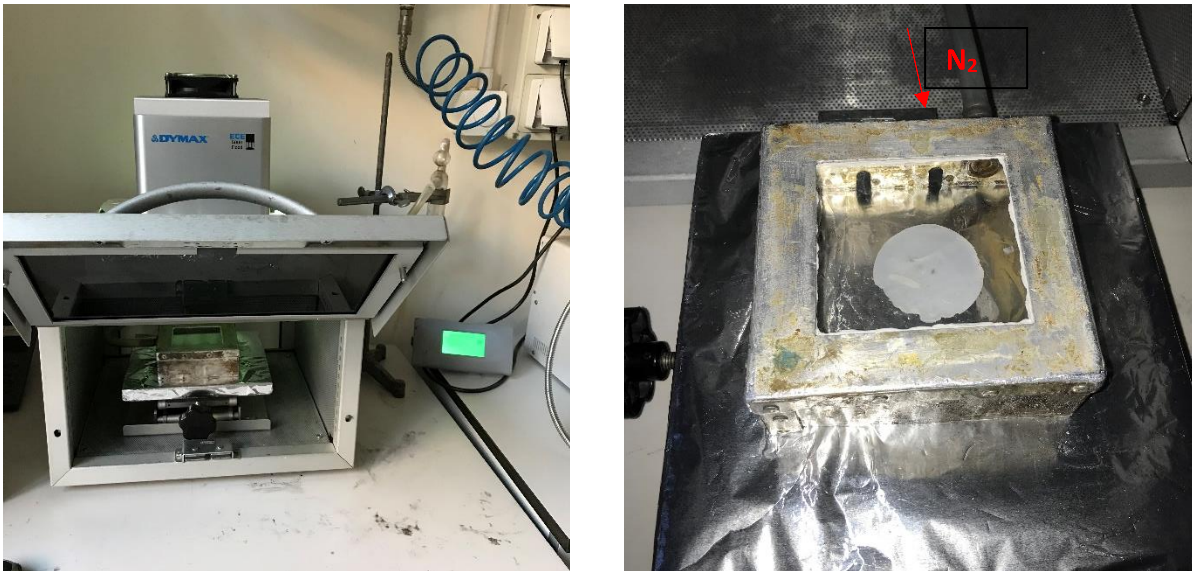

(a) (b)

Figure S1: Photo of (a) 5000-EC UV flood lamp system fitted with nitrogen purged chamber and (b) nitrogen purged chamber with sample inside

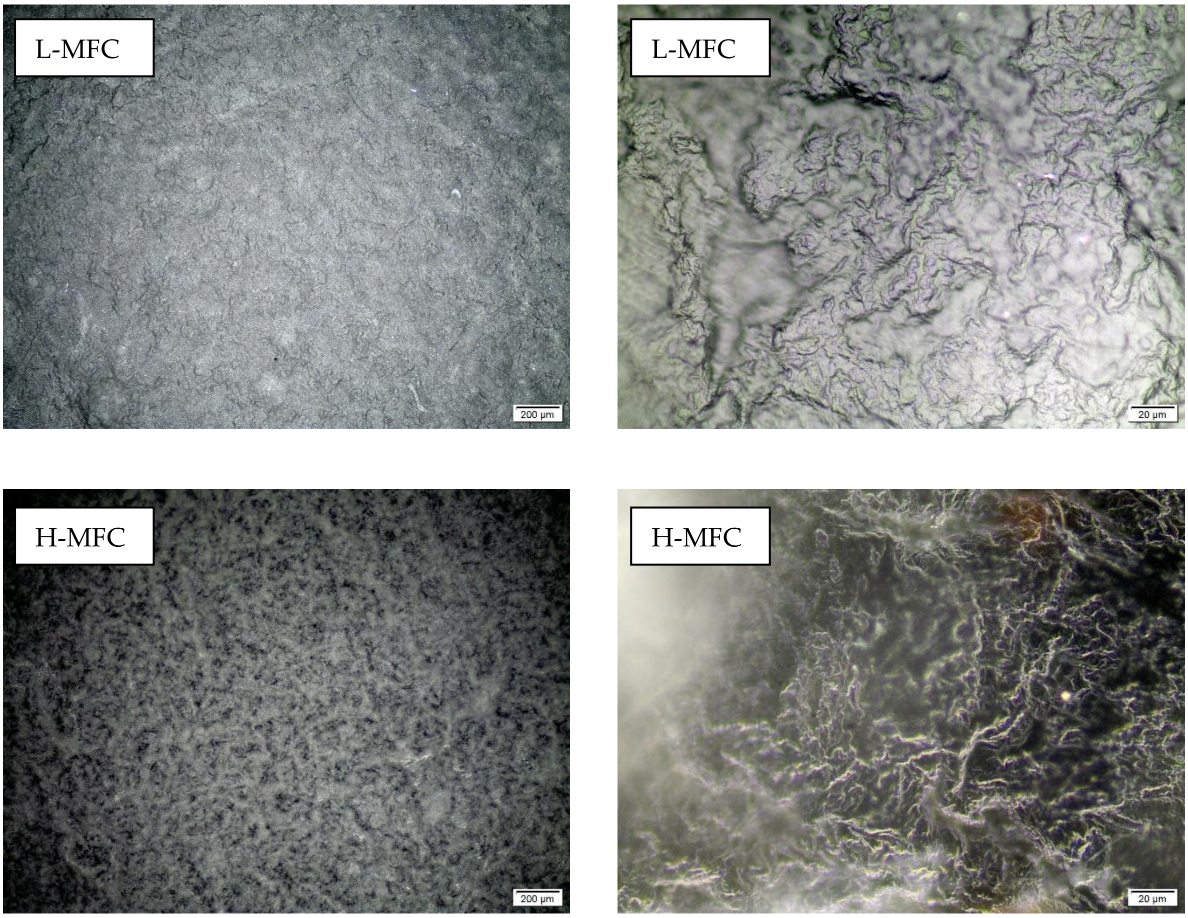

Figure S2: Optical micrographs of the surfaces of composite materials (dark field, in reflection mode)

Optical microscopy was performed in reflection dark field mode with an Olympus BX53M microscope (Olympus Italia S.R.L., Italy).

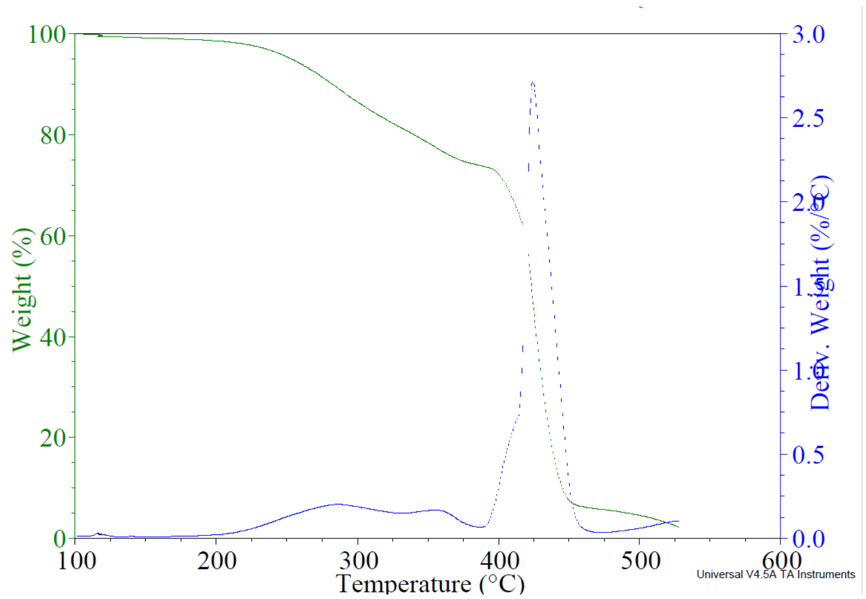

(a)

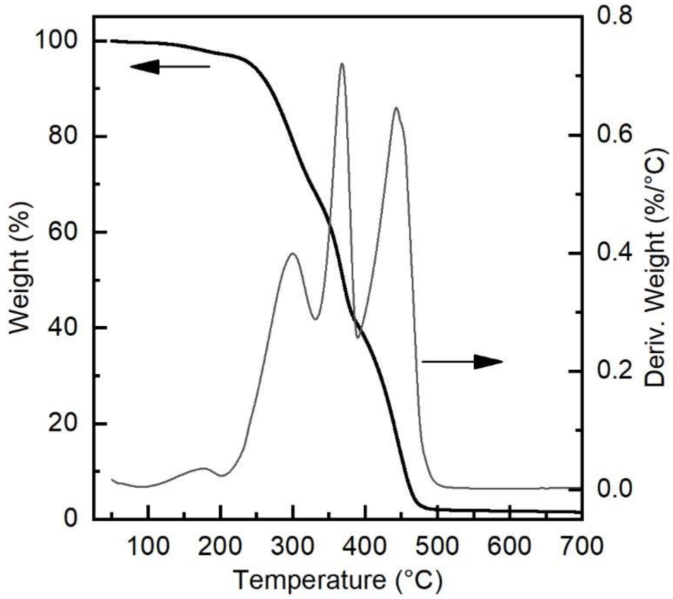

(b)

Figure S3: TGA analysis of (a) CM and (b) L-MFC composite before irradiation

(a) Thermogravimetric analysis of CM was performed on a Q50 TGA from TA Instruments with a temperature ramp of 10 °C min^{-1} from 20 °C to 580 °C under nitrogen flow

(b) Thermogravimetric analysis of L-MFC (before irradiation) was performed on a Q500 TGA from TA Instruments with a temperature ramp of 10 °C min^{-1} from 50 °C to 700 °C under nitrogen flow

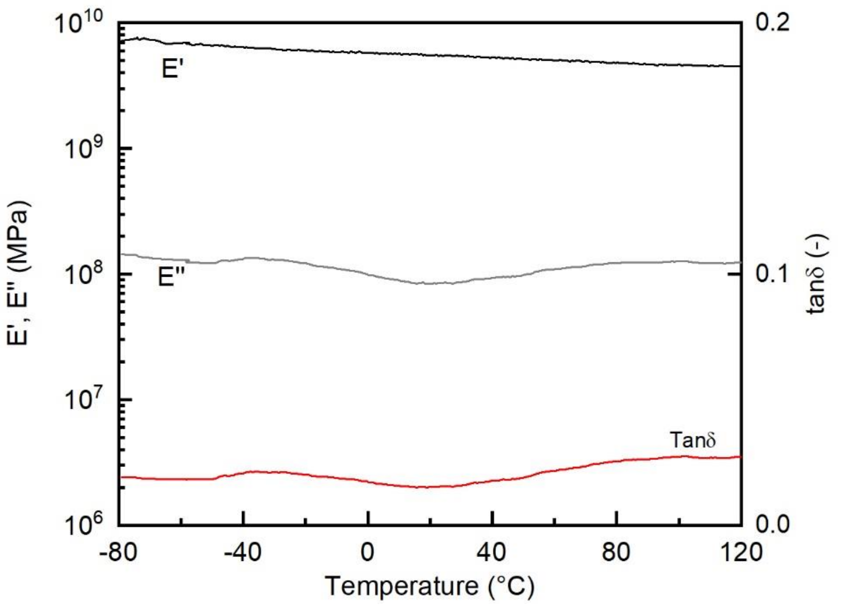

Figure S4. DMA analysis of dry MFC sheet

Dynamic mechanical analysis was performed on a dry MFC sheet with a TTDMA (Triton Technology Ltd., UK) equipment, in the tensile mode. The temperature was increased from -80 °C to 120 °C at a 3 °C min^{-1} rate. The frequency was set at 1 Hz, and the strain was set at 0.01%. The specimens had a length of 10 mm between the clamps, and a width of 6 to 8 mm.

Table S1. Main parameters from TGA and DMA measurements performed on dry MFC, pCM and on photopolymerized L-MFC and H-MFC composites.

Sample	$T_{d,5\%}$	$T_{d,10\%}$	T_{max} (MFC)	T_{max} (Resin)	E′ (-80 °C)	E′ (RT)	Tanδ
MFC	195 °C	314 °C	355 °C	-	7.4 GPa	5.5 GPa	-
pCM	280 °C	308 °C	-	445 °C	-	-	-
L-MFC	205 °C	218 °C	388°C	458°C	4.4 GPa	0.3 GPa	8 °C
H-MFC	205 °C	218 °C	388°C	445°C	3.2 GPa	0.6 GPa	-28 °C
